# Microvascular Obstruction in Acute Myocardial Infarction, a Potential Therapeutic Target

**DOI:** 10.3390/jcm12185934

**Published:** 2023-09-13

**Authors:** Mina Ghobrial, Bilal Bawamia, Timothy Cartlidge, Ioakim Spyridopoulos, Vijay Kunadian, Azfar Zaman, Mohaned Egred, Adam McDiarmid, Matthew Williams, Mohamed Farag, Mohammad Alkhalil

**Affiliations:** 1 Cardiothoracic Centre, Freeman Hospital, Newcastle-upon-Tyne NE7 7DN, UK; 2 Translational and Clinical Research Institute, Newcastle University, Newcastle-upon-Tyne NE1 7RU, UK

**Keywords:** microvascular obstruction, acute myocardial infarction

## Abstract

Microvascular obstruction (MVO) is a recognised phenomenon following mechanical reperfusion in patients presenting with ST-segment elevation myocardial infarction (STEMI). Invasive and non-invasive modalities to detect and measure the extent of MVO vary in their accuracy, suggesting that this phenomenon may reflect a spectrum of pathophysiological changes at the level of coronary microcirculation. The importance of detecting MVO lies in the observation that its presence adds incremental risk to patients following STEMI treatment. This increased risk is associated with adverse cardiac remodelling seen on cardiac imaging, increased infarct size, and worse patient outcomes. This review provides an outline of the pathophysiology, clinical implications, and prognosis of MVO in STEMI. It describes historic and novel pharmacological and non-pharmacological therapies to address this phenomenon in conjunction with primary PCI.

## 1. Introduction

The contemporary definition of successful reperfusion in patients presenting with ST-segment elevation myocardial infarction (STEMI) is shifting towards optimal reperfusion at the microvascular level as well as epicardial flow. Despite advances in primary percutaneous coronary intervention (PPCI), suboptimal microvascular reperfusion remains a significant challenge in patients presenting with STEMI and a source of mortality and morbidity occurring in up to half of PPCIs with seemingly few therapeutic options in routine practice [1,2].

**Figure 1:**
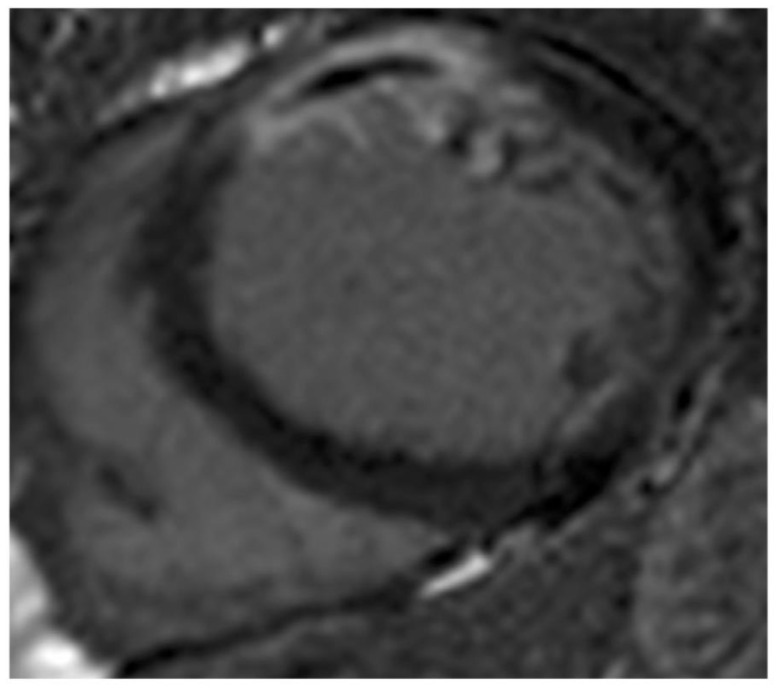
Short axis image illustrating microvascular obstruction (dark) within late gadolinium enhancement myocardium (white) in patient presenting with anterior myocardial infarction.

Early experimental studies highlighted the concept of impaired tissue perfusion despite adequate epicardial flow [3] with subsequent work confirming that the reduction in microcirculatory blood flow is regional and not a global phenomenon [4]. Angiographic slow/no re-flow post percutaneous coronary intervention is defined as Thrombolysis in Myocardial Infarction (TIMI) at a less than 3 flow or a myocardial blush grade (MBG) 0–1 in the infarcted-related artery [5,6]. Failure to achieve ST-segment elevation resolution of more than 70% at one hour after PPCI is also an adverse feature indicating microvascular obstruction [7]. However, the gold-standard test for the evaluation of the extent of microvascular obstruction and resultant infarct zone remains cardiac magnetic resonance (CMR) imaging. MVO appears as a dark focus within an area of late-gadolinium-enhancing myocardium (Figure 1). Indeed, most contemporary clinical trials investigating MVO routinely use CMR to define their endpoints.

## 2. Pathophysiology of MVO

**Figure 2:**
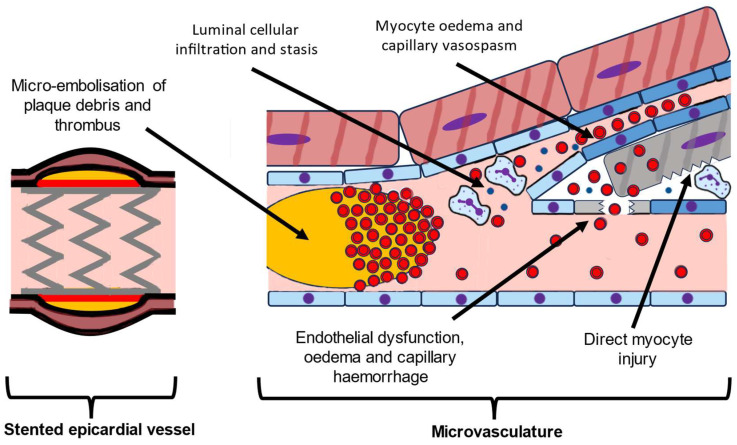
Schematic figure highlighting the pathophysiological process of microvascular obstruction.

The pathophysiology underpinning MVO is a complex interplay between the distal embolisation of thrombus and plaque debris, microvascular vasospasm, endothelial dysfunction, and direct myocardial injury and oedema caused by ischaemia and reperfusion injury (Figure 2). This ultimately results in reduced tissue perfusion and extension of the infarct zone from the endocardium towards the epicardium [5,6].

Some of these elements, such as distal embolization and large thrombus burden, have been directly linked with worse clinical outcomes. In a patient-level meta-analysis, Jolly et al. demonstrated that a high thrombus burden (TIMI thrombus grade ≥ 3) confers an increased risk of cardiovascular death (3.1.% vs. 2.5%; hazard ratio, 0.80; 95% CI, 0.65–0.98; *p* = 0.03) [8]. Similarly, distal embolisation during PPCI and a large residual thrombus burden after PPCI were found to be associated with a higher risk of cardiovascular death (HR 3.63, 95% CI 1.77–7.46, and HR 1.83, 95% CI, 1.13–2.95, respectively) [9,10].

Direct myocardial injury, quantified using mapping techniques, has been linked to large infarct size and extent of MVO [11,12]. MVO is a dynamic phenomenon that regresses over time [13]. Historically, ambiguity over the optimal timing of CMR scanning, the lack of standardised metrics, and varying imaging protocols have made predictions of the extent and severity of myocardial injury more challenging. This variation in practice has limited the assessment of the effectiveness of novel cardioprotective treatments and strategies, and has also been a limitation in comparing results across different studies. Consequently, a consensus document has been proposed to ensure the consistency and standardisation of CMR endpoints in experimental and clinical trials based on pathophysiological changes seen over time [14].

A better understanding of the impact of dysfunctional endothelial cells on microcirculatory function may help to guide novel therapeutic targets to limit MVO. Defective endothelial cells lower the threshold for coronary vasospasm, facilitate thrombogenesis and ultimately undergo apoptosis and fibrotic replacement as a consequence of impaired mitochondrial functions. Endothelial dysfunction is also associated with microvascular degeneration and negative cardiac remodelling in acute myocardial infarction [15]. Furthermore, endothelial dysfunction is not limited to the infarct artery and appears to be a global rather than a regional phenomenon. In fact, microvascular dysfunction, as assessed by invasive coronary physiology studies in non-culprit coronary arteries, was evident in up to 93% of STEMI patients [16]. The relationship between endothelial dysfunction and MVO was also illustrated using a non-invasive modality of peripheral arterial tonometry [17]. Patients with endothelial dysfunction were more likely to develop MVO, larger infarct size, more frequent trans-mural infarcts, and lower ejection fraction [17]. Therefore, the role of endothelial dysfunction post STEMI, particularly in relationship with MVO, should not be underestimated and urgent attention is needed to develop a tailored treatment to address this pathology. Additionally, the role of inflammatory cells in acute myocardial infarction is well established [18,19,20]. Imaging biomarkers have also linked inflammation to plaque instability and the development of acute MI [21,22,23]. Whether inflammation plays a role in exacerbating MVO is yet to be determined [24].

Finally, the inconsistency in detecting MVO using various invasive and non-invasive tools, alongside its complex pathophysiology, adds more challenges to the management of MVO. There was discordance between MVO (detected on CMR) and high-index microcirculatory resistance (IMR, a marker of microvascular dysfunction) in more than one-third of patients presenting with STEMI [25]. MVO could be considered as an anatomical observation related to the pharmacodynamics and distribution of gadolinium contrast agent in patients post STEMI. It merely reflects the ability of the gadolinium contrast agent to reach the infarct core and to be washed out over a pre-defined time (usually 8–10 min). On the other hand, IMR is a functional assessment that directly evaluates the status of microvascular function within the entire distribution of the culprit artery. Notably, there was a near 12-fold increased risk of developing large infarct size at 6 months in patients with MVO and high IMR compared to those with MVO and low IMR [25]. This suggests that MVO reflects a spectrum of pathophysiological processes that are unlikely to respond to a single therapeutic option.

## 3. Prognosis of MVO

Imaging studies have shown that MVO is associated with adverse left ventricular remodelling and increased left ventricular end systolic volumes (LVESV) compared to patients without MVO (change in LVESV index, +4.1 [13.4] vs. −7.0 [12.7] mL/m^2^, respectively, *p* = 0.001) [26]. MVO appears to be a prerequisite for developing intra-myocardial haemorrhagic transformation. The prevailing theory is that intramyocardial haemorrhage (IMH) is a consequence of microvascular rupture, with subsequent extravasation of red blood cells within the infarct core. It is associated with the greatest increases in infarct size and left ventricular volumes as well as the lowest ejection fraction at follow-up [27]. Intramyocardial haemorrhage has also been linked to infarct expansion with a four-fold reduction in myocardial salvage compared to non-haemorrhagic infarcts at follow-up [28]. The myocardial salvage index represents myocardial tissue that was adequately re-perfused and did not transform into necrotic tissue following acute myocardial infarction. It is an established metric that is used to assess the efficacy of treatments in the acute myocardial infarction setting.

Clinical outcomes studies have corroborated imaging data with MVO associated with worse clinical outcomes. For every 1% absolute increase in the extent of MVO, there was a 14% relative increase in 1-year all-cause mortality and an 8% increase in 1-year heart failure hospitalization [1]. MVO remained a significant predictor of all-cause mortality even after adjustment for infarct size [1]. These data, however, do not prove causality and treatment options are urgently needed to reduce MVO and assess whether these options can improve survival or decrease the development of heart failure.

Any strategy to explore the reduction of MVO requires an accurate means of identifying and predicting patients who may subsequently develop MVO at the time of PPCI. The ability to accurately identify such patients would provide an opportunity to test novel strategies at the earliest stage of STEMI management. Certain clinical and angiographic parameters, such as age, high thrombus burden, delayed presentation (symptom to balloon time > 6 h), and pre-stenting IMR, have been identified as potential predictors of MVO [29,30]. Combining these factors in a scoring model provided a reliable method to identify patients at high risk of developing MVO [29,30]. This score was also found to be predictive of intra-myocardial haemorrhage and infarct size, both acutely and at 6 months [29,30].

## 4. Treatments for Microvascular Obstruction

A better understanding of the pathophysiology of MVO enables us to assess treatment options to manage patients at risk of MVO. Numerous treatments for MVO have been studied and are presented in Table 1 and Table 2.

### 4.1. Pharmacological

#### 4.1.1. Antiplatelet Treatment

Suboptimal platelet inhibition around the time of PPCI, assessed by platelet aggregation testing, is relatively common and was observed in almost one-third of patients treated with contemporary dual antiplatelet therapy [31]. Patients with suboptimal platelet inhibition had larger MVO, expressed as a percentage of LV mass, than patients with optimal platelet inhibition (3.2 [IQR, 0.9–5] versus 0.32 [IQR, 0.2–2] respectively; *p* = 0.004) [31]. They were also more likely to sustain major adverse cardiovascular events at 1 year (37% vs. 11%; *p* < 0.01) [31].

The choice of P_2_Y_12_ inhibitor may also impact the extent of MVO. In a study of 128 patients with non-ST segment elevation myocardial infarction (NSTEMI), Xu et al. reported lower pre- and post-PCI IMR in patients treated with ticagrelor compared to clopidogrel (pre-stent IMR 22.0 [13.0–34.9] vs. 27.7 [19.3–29.8]; *p* = 0.02 and post-PCI IMR 22.0 [15.0–29.0] versus 27.0 [18.5–47.5]; *p* = 0.02) [32]. The REDUCE-MVI trial will investigate whether ticagrelor compared to prasugrel may improve microvascular function in patients after revascularized STEMI [33]. Therefore, optimising platelet inhibition may be an effective target for reducing the risk of developing MVO.

Novel agents such as the sodium-glucose cotransporter 2 inhibitors (SGLT2i) have been shown to increase platelet inhibition [34]. The PRESTIGE-AMI (NCT04899479) and EMPRESS-MI (NCT05020704) trials aim to evaluate the impact of SGLT2i on left ventricular remodelling and infarct size using standardised CMR endpoints. Similarly, the PITRI (NCT03102723) trial will investigate the use of cangrelor, an intravenous P_2_Y_12_ inhibitor, in addition to standard aspirin and ticagrelor therapy in patients undergoing PPCI, on LV remodelling, infarct size, and MVO as assessed by CMR.

Glycoprotein IIb/IIIa (GP IIb/IIIa) inhibitors are potent platelet aggregation inhibitors intracoronary (IC) or intravenously (IV) administered. However, there is conflicting evidence of benefits of STEMI. The INFUSE-AMI [35] trial, a blinded, multicentre, randomised control trial, examined the role of IC abciximab and manual aspiration on 452 anterior STEMI patients who underwent PPCI with bivalirudin [36]. Their findings suggested that infarct size as a percentage of total myocardial mass, determined by CMR at 30 days, was significantly lower compared to the placebo group (median, 15.1%; interquartile range [IQR], 6.8–22.7%; n = 181, vs. 17.9% [IQR, 10.3–25.4%]; n = 172; *p* = 0.03). The data on the optimal route to deliver GP IIb/IIIa are even more conflicting. Thiele et al. reported smaller MVO and infarct size comparing IC versus IV abciximab [35]. However, this is in contrast with the AIDA-STEMI trial, the largest randomised trial in this field, which recruited 2065 STEMI patients and failed to show any benefit of IC versus IV abciximab with respect to both composite clinical and CMR endpoints at 1 year [37].

#### 4.1.2. Thrombolytic Therapy

The role of thrombolytic therapy in primary PCI was first described by Sezer et al. who compared low-dose IC streptokinase versus standard therapy immediately after PPCI in 41 patients [38]. The authors observed improved microvascular function in the streptokinase group compared to standard therapy with lower IMR in the treatment group (16.29 ± 5.06 U vs. 32.49 ± 11.04 U) and CFR (2.01 ± 0.57 vs. 1.39 ± 0.31) [38]. However, synthetic tissue plasminogen activators, such as alteplase, have failed to show any improvement in MVO after IC administration following reperfusion but prior to stent implantation. This was described in the T-TIME trial, which randomised 440 patients to varying doses of IC alteplase or placebo [39]. The trial was stopped early due to futility in the interim analysis of the primary endpoint. The investigators reported no difference in the incidence of MVO between the two groups. However, there was a potential signal for harm in relatively late presenters (4–6 h) with an increase in the extent of MVO in the alteplase compared with placebo: 1.14% (placebo) vs. 3.11% (10 mg alteplase) vs. 5.20% (20 mg alteplase); *p* = 0.009 for the trend [40].

Despite these discordant results, it is important to highlight important differences in their designs relating to the thrombolytic agent used and timing of administration, i.e., pre or post stenting. Therefore, further studies are needed to establish the utility (or futility) of using thrombolytic agents as adjunctive treatment in PPCI. The RESTORE-MI (NCT03998319) trial aims to identify patients with MVO based on post-PPCI IMR > 32 and then to randomise to low-dose IC tenecteplase or placebo. The primary endpoints will be a composite clinical outcome and CMR endpoints that include MVO and intramyocardial haemorrhage. Similarly, the RECOVER II (NCT04571580) trial will assess low-dose reteplase as adjunctive therapy in patients with anterior STEMI, after reperfusion but prior to stent implantation, with CMR infarct size as the primary endpoint.

#### 4.1.3. Intracoronary Vasodilators

In clinical practice, adenosine, epinephrine, sodium nitroprusside (SNP), and verapamil have been used as vasodilator agents to help reduce MVO and no-reflow. The evidence to support their use is limited given the small sample sizes, the heterogeneity of administered doses, and the lack of CMR endpoints. The REFLO-STEMI trial compared the use of SNP or adenosine to standard treatment and showed no reduction in MVO or infarct size on CMR with either treatment [41]. There was a signal of harm, defined as a larger infarct size compared to the control, with routine use of adenosine pre and post stenting [41].

On the other hand, intra-coronary epinephrine showed better efficacy and improvement in TIMI flow, MBG, and resolution of ST-segment elevation with a signal for reduction in adverse clinical outcomes at 30 days [42,43,44]. A recent network meta-analysis of 41 randomised control trials (RCTs) and 4069 patients suggested that anisodamine, a muscarinic cholingeric antagonist, and SNP improve coronary flow and MACE outcomes compared to other IC agents or control groups [45].

#### 4.1.4. Intensive Statin Therapy

High-dose statins immediately administered prior to PPCI were associated with improved TIMI flow, MBG, and ST-segment resolution compared to those who received them post-PPCI [46]. The underlying mechanisms behind these observations are unclear, but could be attributed to the pleotropic effects of statins in improving endothelial function, increasing nitric oxide bioavailability, antioxidant and anti-inflammatory properties, and plaque stabilization [47]. However, the modest effect size of statins in reducing no-reflow limits generalisability of using high-dose statins in the treatment of MVO. Despite this, incorporating pre-medication with high-dose statins in addition to standard care should be encouraged as a potentially economical and efficacious means of preventing MVO in STEMI.

## 5. Non-Pharmacological

### 5.1. Thrombectomy

Results in reference to the use of thrombectomy aspiration catheters in STEMI are variable. Early randomised clinical trials reported angiographic benefits when using manual thrombus aspiration compared to routine PCI, which translated into improved clinical outcomes at 30 days [48]. However, subsequent randomised clinical trials failed to show clinical benefit or infarct size reduction following routine thrombectomy [35,49,50]. However, some studies have suggested that a selective approach of using aspiration catheters such as in patients with larger thrombus burden may prove effective [8,10]. It is yet to be determined whether the selective use of better manual thrombectomy tools may help improve microvascular function and, subsequently, clinical outcomes.

Nevertheless, mechanical thrombus aspiration using the AngioJet^TM^ (Boston Scientific, MA, USA) rheolytic thrombectomy system was not associated with a reduction in infarct size or mortality at 30 days [51]. The CHEETAH study reported excellent angiographic results when using the Indigo CAT RX Aspiration System (Penumbra Inc., Alameda, CA, USA) [52]. In this single-arm study of 400 patients, the Penumbra mechanical aspiration catheter creates a continuous and sustained vacuum leading to TIMI 3 flow in 97.5% of cases and MBG 3 in 99.75% [52]. Recently, stent retrieval technology has provided an alternative solution when treating STEMI patients with large thrombus burden [53]. Future studies, such as the NATURE trial, would add further insights into the role of clot retrievals in managing MVO.

### 5.2. Selective Intracoronary Hypothermia (SIH)

Selective intracoronary hypothermia hypothesizes that rapidly and selectively cooling the infarct territory to approximately 6 °C below body temperature prior to reperfusion will mitigate reperfusion and/or ischaemic injury whilst avoiding the delays, inadequacy, and deleterious effects of systemic hypothermia [54]. Reperfusion injury plays an important role in myocardial infarction with subsequent changes at the level of the myocardium, including oedema, and intra-myocardial haemorrhage [11,13]. Selective hypothermia is performed by means of an over-the-wire (OTW) balloon with 4 °C saline infusion during OTW balloon occlusion prior to full mechanical reperfusion [54]. By reducing reperfusion injury, this may attenuate MVO and reduce the risk of IMH.

The EURO-ICE trial (NCT03447834) is a prospective, multicentre, randomised proof-of-concept trial designed to ascertain the impact of SIH against the standard of care in anterior STEMI patients. It will assess CMR-derived infarct size at 3 months and composite clinical endpoints at 3 months and 1 year. The safety of this treatment has been reported using the first 50 patients recruited compared with the first 50 patients randomised to the control arm of the trial [55]. In-hospital mortality was 0% with no differences between groups with respect to rhythm or conduction disturbances, stent thrombosis, or onset of heart failure during the procedure [55].

### 5.3. Pressure-Controlled Intermittent Coronary Sinus Occlusion (PiCSO^®^)

PiCSO^®^ (Miracor Medical SA, Brussels, Belgium) is a pressure-controlled balloon catheter placed in the coronary sinus during PPCI. It intermittently increases mean coronary sinus pressure and coronary sinus pulse pressure to maintain microcirculatory patency, redistributing blood flow to areas of ischemic myocardium [56]. Non-randomised studies did not report significant differences in the extent of MVO or IMH, despite a reduction in post-PCI IMR in anterior STEMI patients treated with PiCSO^®^ compared to standard therapy [57,58]. However, the final infarct size at 6 months was reduced in PiCSO^®^ patients as assessed by CMR imaging (26.0% [20.2–30.0] vs. 33.0% [28.0–37.0], *p* = 0.006) [57,58].

The PiCSO-AMI-1 (NCT03625869) trial will be the first randomised trial exploring the safety and efficacy of PiCSO^®^ in patients presenting with anterior STEMI and TIMI 0 or 1 compared to standard therapy.

### 5.4. Left Ventricular Unloading

Animal studies have demonstrated that left ventricular unloading prior to reperfusion reduces infarct size by 40–50% [59]. The proposed mechanism was related to the reduction in myocardial oxygen consumption and improvement in microcirculatory perfusion [59]. However, studies assessing the role of the intra-aortic balloon pump (IABP) failed to improve clinical outcomes [60]. The advent of Impella^®^ (Abiomed Inc., Danvers, MA, USA), a transvalvular axial flow-pump, which is currently used in high-risk PCI and cardiogenic shock, has renewed interests in LV unloading in the setting of STEMI without cardiogenic shock [60]. In a pilot study of 50 patients with anterior STEMI without cardiogenic shock randomised to either LV unloading with Impella-CP^®^ followed by immediate reperfusion or LV unloading for 30 min prior to reperfusion, there was no safety signal in delaying reperfusion by 30 min [61]. Importantly, this study lacked a control group, whereby patients underwent standard PPCI, to assess the potential harm of delaying reperfusion by 30 min [61]. Additionally, the total ischemia time between the two groups was comparable, making interpretation of the study results more challenging [61]. Any future study would need to assess the overall bleeding rate given that the rate of Bleeding in Academic Research Consortium (BARC) ≥ 2 was observed in up to 16% of patients in the trial [61]. Nonetheless, the results of this pilot study have informed the design of the DTU-STEMI trial (NCT03947619), which is currently recruiting anterior STEMI patients without cardiogenic shock to LV unloading with Impella CP^®^ for 30 min followed by reperfusion or conventional PPCI. The primary endpoint is infarct size on CMR. Whether unloading the left ventricle will ameliorate reperfusion injury and improve MVO is yet to be determined.

### 5.5. Sonothrombylysis

Sonothrombolysis is a novel strategy in managing STEMI patients achieved by inducing microbubble cavitation using high-mechanical index ultrasonic impulses from conventional transducers after the infusion of intravenous ultrasound-enhancing agents [62]. A small, randomised clinical trial involving 100 STEMI patients demonstrated promising results, with reduced infarct size following sonothrombolysis when compared to standard PPCI and improved LV ejection fraction at six months [63]. Although the extent of MVO was not different between the two groups, there was a tendency towards a lower degree of MVO in patients presenting with anterior STEMI who underwent sonothrombolysis [63]. Further studies are needed to confirm these findings.

### 5.6. Supersaturated Oxygen (SSO_2_)

Supersaturated oxygen therapy provides oxygenated blood with a partial arterial pressure (PaO_2_) between 760 and 1000 mmHg to the left coronary artery through a coronary catheter after PPCI [64]. Different mechanisms were proposed to rationalise the use of SSO_2_ in patients presenting with STEMI. Experimental studies suggested that SSO_2_ decreased tissue oedema, reduced the formation of lipid peroxide radicals, and inhibited inflammatory cell adherence, leading to improved coronary microcirculation [64,65].

Previous randomsied clinical trials reported smaller infarct sizes in patients presenting with anterior STEMI and subjected to supersaturated oxygen [65,66]. However, there was a signal of increased stent thrombosis in patients receiving SSO_2_, which may be related to the position of the delivery catheter within the stented segment [65,66]. A recent study demonstrated the safety and feasibility of delivering SSO_2_ to the left main artery, remote from the stented segment [67].

### 5.7. Deferred Stenting

The potential advantage of deferred stenting in STEMI is to avoid slow-flow or no-reflow with subsequent deleterious effects on microvasculature, thereby improving myocardial salvage and reducing infarct size [68]. Previous studies have suggested that deferred stenting was associated with a reduction in infarct size and improved myocardial salvage, resulting in better clinical outcomes in selected patients [69,70,71]. This included patients with a high-thrombus burden, late-presenters, or with long segments of disease requiring long stents (>24 mm) [69,70,71]. Additional benefits included the avoidance of angioplasty in up to 38% of patients and larger stent diameters with a low rate of IRA re-occlusion [69,70,71].

It is important to note that prolonged GP IIb/IIIa infusion is a corner-stone of this strategy, reflecting the near 100% usage in most of these studies. However, conflicting data on the efficacy of this routine use combined with the optimal duration of the deferral strategy remain unclear [71,72]. The LATE (NCT02445885) and OPTIMAL (NCT03282773) randomised trials will look to explore the effects of deferred stenting in late-presenters (>12 h) and left main stem STEMI, respectively.

## 6. Conclusions

The presence of MVO after STEMI is associated with increased infarct size, adverse LV remodelling, and poor patient outcomes, and its management remains challenging. A better understanding of the mechanistic processes contributing to MVO would enable us to design tailored treatments for its underlying complex and multifactorial pathophysiology. A reliable and accurate diagnostic and/or imaging modality would allow the characterization and identification of patients most likely to benefit from intervention. A variety of innovative pharmacological and device-based therapies have been and are currently being tested and the best approach may be an integrated approach of some of these promising interventions.

The delayed impact of MVO on morbidity and mortality is a burden on patients, their carers, and healthcare providers underscores the need for further research in the prevention, diagnosis, and treatment of this condition.

## Figures and Tables

**Table 1:** A summary of pharmacological interventions for microvascular obstruction.

Intervention	Efficacy ^(a)^	Current Level of Evidence ^(b)^	Ongoing RCTs
**Pharmacological**			
High-dose Statins	++	A	No
IC/IV Glycoprotein IIb/IIIa inhibitors	++	A	No
IC Thrombolytics	−	B	Yes
IC Epinephrine	+	B	No
IC Anisodamine	+	B	No
IC SNP	+	B	No
IC Adenosine	+/−	B	No
IC Non-DHP CCBs	+/−	B	No
IC Cangrelor	Unknown	C	Yes
SGLT2 inhibitors	Unknown	C	Yes

IC (intracoronary) IV (intravenous), Non-DHP CCBs (non-dihydropyridine calcium channel blockers), SNP (Sodium nitroprusside), SGLT2i (sodium-glucose cotransporter 2 inhibitor). + (some efficacy), ++ (moderate efficacy), +/− (equivocal efficacy), − (potentially harmful). ^(a)^ Efficacy of the intervention is based on the authors’ subjective interpretation of the data based on the quality and magnitude of the end-point change according to the currently available data which is subject to change with future research. ^(b)^ Level of evidence.

**Table 2:** A summary of non-pharmacological interventions for microvascular obstruction.

Intervention	Efficacy ^(a)^	Current Level of Evidence ^(b)^	Ongoing RCTs
**Non-Pharmacological**			
Routine Manual Thrombectomy	−	A	No
Mechanical Thrombectomy	+/−	B	No
Selective Intracoronary Hypothermia	Unknown	C	Yes
PICSO^®^	++	B	Yes
Sonothrombolysis	++	B	Yes
Left Ventricular Unloading	+	B	Yes
Supersaturated Oxygen	++	B	No
Deferred Stenting	+/−	A	Yes

+ (some efficacy), ++ (moderate efficacy), +/− (equivocal efficacy), − (potentially harmful). ^(a)^ Efficacy of the intervention is based on the authors’ subjective interpretation of the data based on the quality and magnitude of the end-point change according to the currently available data which is subject to change with future research. ^(b)^ Level of evidence.

## Data Availability

Not applicable.
